# Evaluation of the relationship between serum interleukin-1β levels and expression of inflammasome-related genes in patients with COVID-19

**DOI:** 10.1186/s12865-023-00568-x

**Published:** 2023-09-18

**Authors:** Zahra Bagheri-Hosseinabadi, Ali Shamsizadeh, Fatemeh Bahrehmand, Mitra Abbasifard

**Affiliations:** 1 Immunology of Infectious Diseases Research Center, Research Institute of Basic Medical Sciences, Rafsanjan University of Medical Sciences, Rafsanjan, Iran; 2 Department of Clinical Biochemistry, School of Medicine, Rafsanjan University of Medical Sciences, Rafsanjan, Iran; 3 Physiology-Pharmacology Research Center, Research Institute of Basic Medical Sciences, Ali-Ibn Abi-Talib Hospital, Rafsanjan University of Medical Sciences, Rafsanjan, Iran; 4 Department of Internal Medicine, Ali-Ibn Abi-Talib Hospital, School of Medicine, Rafsanjan University of Medical Sciences, Rafsanjan, Iran

**Keywords:** Coronavirus disease 2019, Severe acute respiratory syndrome coronavirus 2, Inflammasome, IL-1β, NLRP1, NLRP3

## Abstract

**Background:**

Inflammasomes are a group of molecules that are strongly involved in causing inflammation. This study aimed to evaluate the expression of NLR family pyrin domain containing 1 (NLRP1), NLRP3, and Apoptosis-associated speck-like protein containing a CARD (ASC) as well as their association with serum level of interleukin (IL)-1β in patients with coronavirus disease 2019 (COVID-19).

**Methods:**

Thirty COVID-19 patients and 30 healthy subjects (HS) were recruited. Peripheral blood specimens were collected from subjects to assess NLRP1, NLRP3, and ASC gene expression by Real time-PCR technique. Serum levels of IL-1β were also measured via the enzyme-linked immunosorbent assay (ELISA).

**Results:**

The findings showed no significant differences in serum IL-1β level between COVID-19 patients and the HS group. mRNA expression of ASC (*P* = 0.008) and NLRP1 (*P* = 0.03) gene had a significant increase in COVID-19 patients compared to HS, while there was no significant increase in the expression of NLRP3 between the studied group. There were significant correlations between patient’s data and expression levels of NLRP1, NLRP3, IL-1β, and ACS.

**Conclusions:**

NLRP1 and ASC may have a more critical role in the generation of the active form of IL-1β in COVID-19 patients compared to NLRP3. However, serum levels of IL-1β in patients did not show a significant increase, which may be due to the patient’s condition and the application of virus escape mechanisms through impaired NLRP3 expression and its malfunction.

## Introduction

Our knowledge of coronaviruses to date shows that they can cause respiratory tract infection in humans with different clinical manifestations associated with dysregulated immune system inflammatory responses [1]. Severe acute respiratory syndrome coronavirus 2 (SARS-CoV-2), known as a β-coronavirus that was first identified in Wuhan, Hubei province, China, in late 2019, spread around the world rapidly [1, 2]. Although the virus often causes the usual and tolerable flu-like symptoms, in some people, it causes severe immune-mediated reactions such as cytokine storms which could be associated with damage to tissues such as the lungs, kidneys, brain, and cardiovascular system [3].

Evidence showed that in patients who react severely to the SARS-CoV-2, elevated serum levels of inflammatory cytokines such as IL-1β, IL-6, IL-8, and IL-18 could be detectable [4]. Therefore, inflammatory cytokines appear to be one of the most critically involved immune-mediators in the pathogenesis of COVID-19 and associated disorders [5]. It has been shown that identifying pathogen-associated molecular patterns (PAMPs) derived from SARS-CoV-2 by pattern-recognition receptors (PRRs) of innate immunity can lead to activation of inflammasomes as cytosolic multi-protein oligomers that are responsible for the stimulation of innate inflammatory responses [6]. The essential molecules in the inflammasome biostructure are NLRP1 and NLRP3, which can stimulate ASC and caspase-1, resulting in the generation of the active form of IL-1β [7].

Furthermore, releasing of IL-1β can have several inflammatory consequences, including participation in the development of cytokine storm and infectious shock [8, 9]. Recent studies have shown that following SARS infection detection by innate immune system components and receptors, NLRP1 and NLRP3 inflammasomes become activated and can produce inflammatory cytokines such as IL-1β and IL-18 [10, 11]. However, the precise role of molecules involved in the formation of inflammasomes and IL-1β in causing cytokine storms during COVID-19 infection is unknown.

Given the growing body of evidence linking dysregulated immune responses and cytokine storm to severe COVID-19 outcomes, investigating the gene expression of NLRP1, NLRP3, and ASC, as well as the serum levels of IL-1β, in patients with COVID-19 is of paramount importance. By deciphering the intricate interplay between these molecular components, we can gain insights into the mechanistic underpinnings of hyperinflammation and its potential contribution to disease severity.

## Materials and methods

### Subjects

Thirty COVID-19 patients were involved in this case-control cross-sectional study that were recruited from Ali-Ibn-Abitaleb Hospital, Rafsanjan, Iran during the first 6 months of 2022. Furthermore, 30 age and sex-matched healthy subjects (HS) were selected. Positive RT-PCR test, hospitalized, radiological evidence, and other associated clinical observations considered as inclusion criteria for COVID-19 patients. Furthermore, all participants with other viral infections, respiratory system-related illnesses, allergies, autoimmunity, malignancies, and immunocompromised subjects were excluded from the investigation. The demographic and clinical data of the participants, including age, sex, duration of the hospitalization, type of medication before and during the SARS-CoV-2 infection, disease outcome (discharge, death), duration of symptom onset, the severity of the symptoms of COVID-19 patients (CT scan and chest x-ray findings), and vital signs of patients were recorded and documented. Moreover, blood laboratory parameters, including lymphocyte and neutrophil count, levels of C-reactive protein (CRP), erythrocyte sedimentation rate (ESR) were measured and recorded. To perform all experiments, about 5 mL of peripheral blood was obtained form study participants using venipuncture in tubes containing EDTA and clot activator tubes. Informed consent was obtained from each COVID-19 patient and HS based on the Declaration of Helsinki and the Ethics Committee of Rafsanjan University of Medical Sciences, Rafsanjan, Iran was approved this investigation (IR.RUMS.REC.1399.021).

### Blood analysis

Blood samples were collected from studied groups and divided into three fractions due to serum separation and ELISA, cell blood count, as well as total RNA extraction. In the following, the serum samples were stored at -20 ° C until further experiments.

### IL-1β assay

To measure the IL-1β serum levels, a commercial (Karmania Pars Gene Company, Iran) ELISA kit was used, and the procedure was completed according to the manufacturers’ instructions. Based on the kit information, the assay range and sensitivity were 0.2 pg/mL-15 pg/mL, respectively. The outcomes were only considered for further analysis when inter-and intra-assays values were CV < 15% and CV < 5%, respectively.

### Gene expression assay

According to the manufacturer’s instruction, total RNA was extracted from whole blood cells by Karmania pars gene RNA extraction kit in this study. The extracted RNAs’ purity and integrity were assessed using spectrophotometric method (NanoDrop ND-2000, Thermo Fisher) with the calculation of 260/280 ratio. Next, cDNA templates were synthesized using Karmania pars gene one-step cDNA synthesis kit by 15µL ready to use cDNA master mix and a 5µL of 1ng to 5 µg RNA template as per protocol recommended by the manufacturer: 42–50 °C for 30 min; 90 °C for 5 min; (reverse transcriptase (RT) enzyme inactivation), and lastly the microtubes were chilled on the ice for 2 min. Additionally, specific sense and antisense primers (0.5 µM) (Table 1), 2X qPCRBIO SYGreen Mix Hi-ROX (PCRBiosystem, England), and nuclease-free water were used to amplification by a Rotor-Gene Q 2plex System (Qiagen) according to the suggested protocol: 1 cycle of 95 °C for 2 min; 40 cycles of 95 °C for 5 s (Denaturation), and 60–65 °C (annealing/Extension) for 20 to 30 s. Moreover, the melting curve step was considered for the final step by 10 s at 95 °C and then 10 s each at 0.2 °C enhancements between 62 and 95 °C. The RT-PCR was done in triplicate, and GAPDH was used as the reference gene to normalize the obtained signals. Ultimately, the relative expression of the PCR products was calculated by the 2^−ΔΔCt^ formula.

**Table 1:** The sequences of primers used in the study

Gene	Sense	Antisense
**GAPDH**	CCAGAACATCATCCCTGCCT	CCTGCTTCACCACCTTCTTG
**NLRP1**	GACGCCGCATTGACCATCTA	CTCCTTCAGGTTTCTGGTGACC
**NLRP3**	GGACTGAAGCACCTGTTGTGCA	TCCTGAGTCTCCCAAGGCATTC
**ASC**	CTGGAGCCATGGGGCGCGCG	CGGAGTGTTGCTGGGAAGGAG

*GAPDH* Glyceraldehyde 3-phosphate dehydrogenase, *NLRP* NLR family pyrin domain containing

### Statistical analysis

GraphPad Prism 9 (GraphPad Software, San Diego, CA) was used to perform statistical analysis. The Shapiro–Wilk test was employed to evaluate variables’ normality of distribution. The studied groups’ differences were calculated using the independent sample T-test and Mann–Whitney U test. Moreover, to estimate the relationship between patients’ data and gene expression, the correlation matrix test was used. All data are presented as mean ± SEM, and a *P* value less than 0.05 was considered statistically significant.

## Results

### Participants’ data

A total of 60 participants, including 30 patients with COVID-19 and 30 HS joined in this study. Patient group was comprised of 16 (53.34%) male and 14 (46.66%) female subjects, while the HS group involved 15 (50%) males and 15 (50%) females. Mean age of the patients and HS was 65.93 ± 2.44 and 64.28 ± 3.45 years, respectively. There were 8 (26.67%) and 5 (16.67%) smoker subjects in patient and HS groups, respectively. In COVID-19 group, while 8 (26.67%) cases had sever disease form, 22 (73.33%) subjects had mild disease intensity. Upon following up, 29 (96.67%) patients discharged while 1 (3.33%) patient expired. Patients’ data is listed in Table 2.

**Table 2:** Clinical and laboratory data of studied patients with COVID-19

COVID-19 patient (*n* = 30)	WBC/(µL)	PLT/(µL)	Neutrophil count /(µL)	Lymphocyte count /(µL)	CRP (mg/mL)	O2 (mm Hg)	Fever (°C)	Medication used	Age (Years)	Duration of hospitalization (Days)
1	3900	176	65	33	55	80	38	Hydroxychloroquine sulfate, interferon beta, Tamiflu-oseltamivir, methylprednisolone	91	15
2	5900	166	69	19	9	84	38.9	Hydroxychloroquine sulfate, interferon beta, Tamiflu-oseltamivir, methylprednisolone	65	31
3	6500	229	71	34	50	86	38.5	Lopinavir & ritonavir (Kaletra ®)	61	9
4	7300	342	77	20	8	94	38	Hydroxychloroquine sulfate	65	6
5	7000	211	65	33	6	93	37	Hydroxychloroquine sulfate	38	8
6	4100	165	60	37	53	92	37.5	Lopinavir & ritonavir (Kaletra ®), Tamiflu-oseltamivir, hydroxychloroquine sulfate	79	11
7	4100	152	52	39	32	96	36.6	Lopinavir & ritonavir (Kaletra ®), Tamiflu-oseltamivir, hydroxychloroquine sulfate	70	6
8	5300	140	72	21	30	92	37	Interferon beta, hydroxychloroquine sulfate, methylprednisolone	50	8
9	3800	342	46	50	55	95	37	Hydroxychloroquine sulfate	56	3
10	`4500	157	63	25	10	93	37.2	Hydroxychloroquine sulfate	60	5
11	4500	221	70	28	6	98	37	Hydroxychloroquine sulfate	88	3
12	2300	210	61	31	50	97	36.4	Interferon beta, hydroxychloroquine sulfate, methylprednisolone	58	8
13	5100	205	65	29	24	89	38	Hydroxychloroquine sulfate, methylprednisolone	56	3
14	2300	195	67	31	6	85	37.2	Interferon beta, hydroxychloroquine sulfate, methylprednisolone	70	15
15	5400	95	75	19	16	92	37	Interferon beta, lopinavir & ritonavir (Kaletra ®), selenium, methylprednisolone, dexamethasone	56	26
16	4200	215	70	30	35	95	38	Interferon beta, selenium, methylprednisolone	61	15
17	3100	242	64	30	8	87	36.2	Lopinavir & ritonavir (Kaletra ®)	63	6
18	5000	181	55	40	9	88	37.5	Lopinavir & ritonavir (Kaletra ®), Tamiflu-oseltamivir, hydroxychloroquine sulfate	63	6
19	7300	225	79	20	24	90	37	Interferon beta, hydroxychloroquine sulfate, methylprednisolone	59	8
20	8300	190	70	25	25	90	38	Hydroxychloroquine sulfate	52	5
21	1380	259	87	11	16	89	40	Tamiflu-oseltamivir, hydroxychloroquine sulfate, lopinavir & ritonavir (Kaletra ®)	65	7
22	1900	67	78	22	20	94	37	Interferon beta, methylprednisolone	88	11
23	1180	288	65	25	127	92	37	Interferon beta, dexamethasone	77	4
24	4600	220	75	25	15	80	38.9	Interferon beta	70	8
25	6100	150	85	13	42	95	37.5	Dexamethasone, interferon beta	61	7
26	7000	174	86	12	21	92	37.9	Interferon beta, methylprednisolone	67	8
27	3500	183	70	30	14	75	37.2	Interferon beta, dexamethasone	96	9
28	5300	215	60	37	51	99	37	Interferon beta, methylprednisolone	45	9
29	6600	222	79	16	20	87	37.5	Interferon beta, hydroxychloroquine sulfate, methylprednisolone	75	15
30	5300	105	82	16	20	66	37	Interferon beta, lopinavir & ritonavir (Kaletra ®), hydroxychloroquine sulfate, dexamethasone	73	11
**All (mean ± SEM)**	**4768 ± 347.8**	**198.1 ± 11.2**	**69.43 ± 1.8**	**26.70 ± 1.67**	**28.57 ± 4.53**	**89.5 ± 1.3**	**37.5 ± 0.14**		**65.93 ± 2.44**	**9.533 ± 1.14**

### Gene expression quantification

**Fig. 1:**
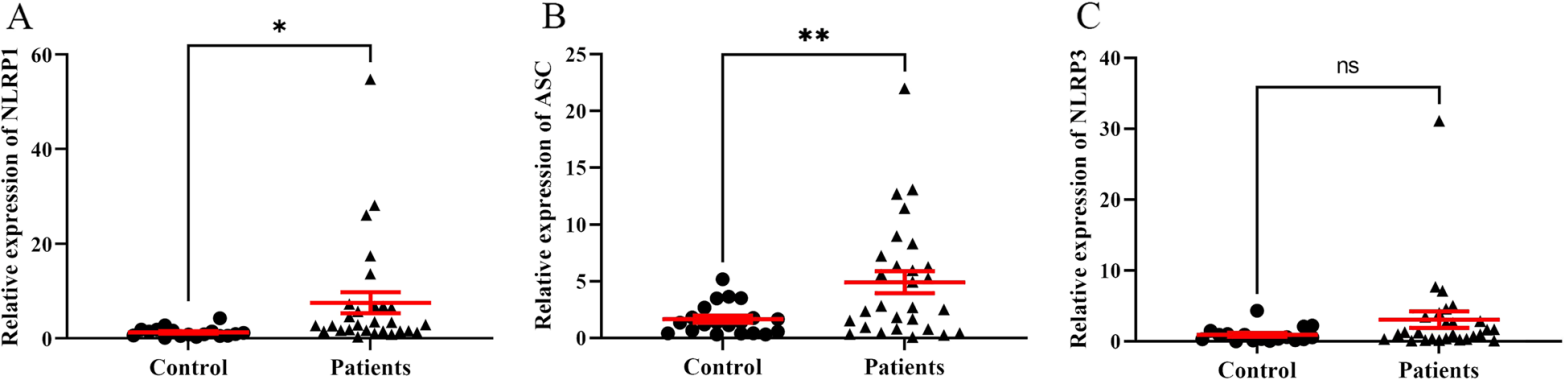
Relative expression of NLRP1 (**A**), ASC (**B**), and NLRP3 (**C**) in control group (HS) and patients with COVID-19. All tests were performed in triplicate. Data are presented as mean ± SEM. *P < 0.05, ** *P*˂ 0.01, ns: non-significant, HS: healthy subjects

**Fig. 2:**
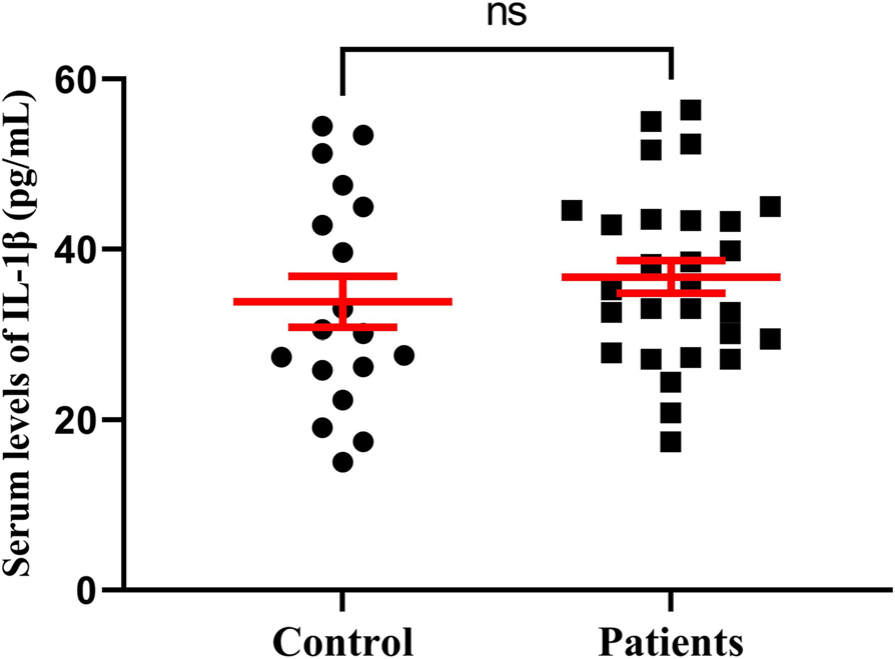
The differences between serum levels of IL-1β in control (HS) and COVID-19 patients. Data are presented as mean ± SEM; A *P*-value < 0.05 was considered statistically significant. Ns: non-significant, HS: healthy subjects

Findings of the expression of the studied genes showed that NLRP1 and ASC genes had a significant up-regulation in the patient group compared to the HS group (*P* = 0.03 and *P* = 0.008, respectively). While the NLRP3 mRNA level had an up-regulation in the COVID-19 patients compared to HS group, this elevation was not statistically significant (*P* = 0.16; Fig. 1). Also, the serum level of IL-1β in the patient group had not significant changes compared to the HS group (*P* = 0.34; Fig. 2).

### Correlation analysis

**Fig. 3:**
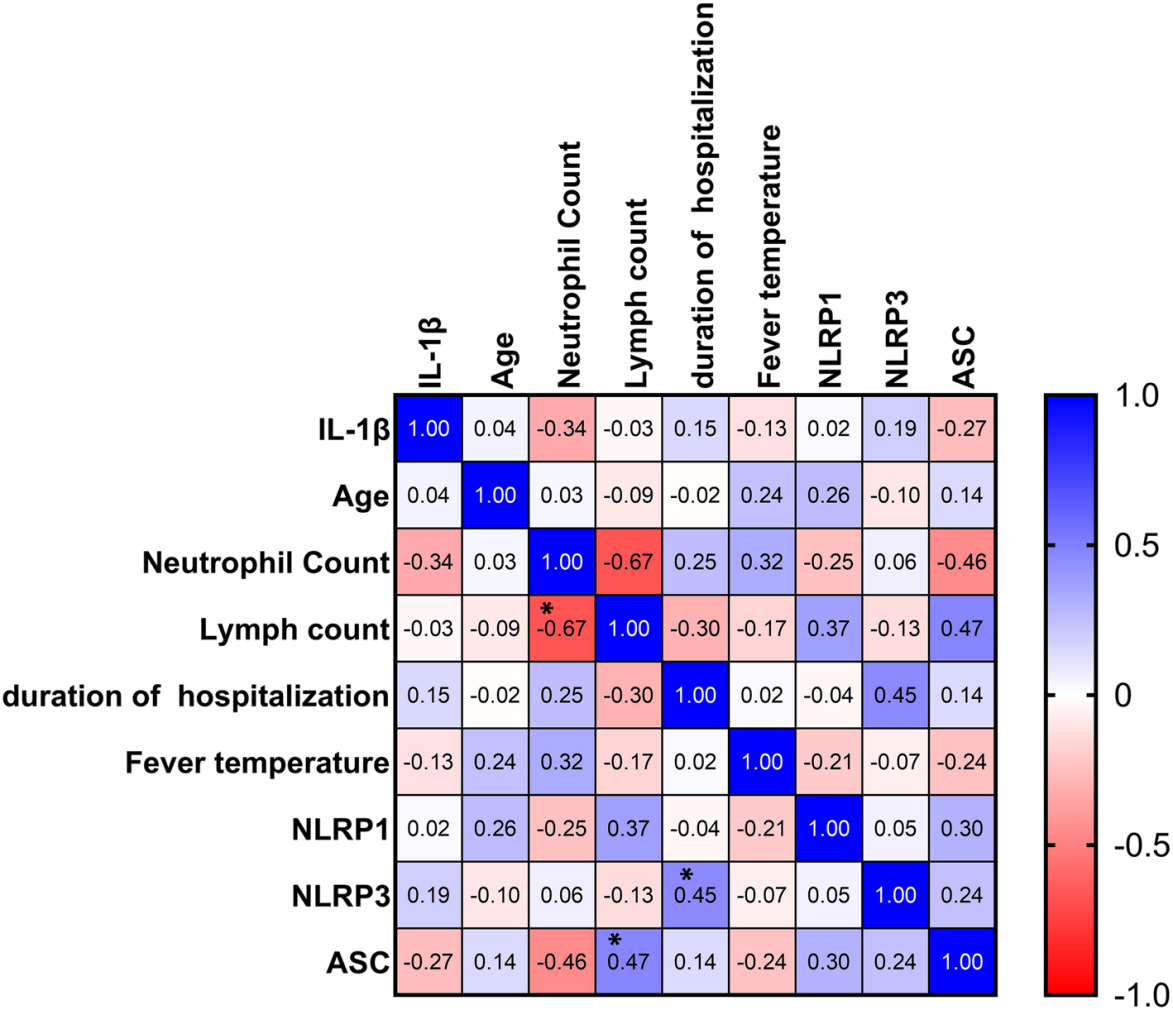
The correlations between the expression of NLRP1, NLRP3, ASC, IL-1β serum level, and other related variables in patients with COVID-19. The correlation coefficient is shown inside each square (*P ˂ 0.05)

The statistical analysis results showed a significant and negative correlation between neutrophil count and lymphocyte count in patients with COVID-19 (*r*=-0.67, *P* < 0.0001). There was a significant and positive correlation between the expression of NLRP3 and the duration of hospitalization (*r* = 0.45, *P* = 0.018). Also, there was a significant and positive association between the expression of ASC and lymphocyte count (*r* = 0.47, *P* = 0.012), whereas the association between the expression of this molecule and neutrophil count was negative and significant (*r*=-0.46, *P* = 0.016; Fig. 3).

## Discussion

The latest evidence suggested that increased and uncontrolled inflammation plays a pivotal role in the pathogenesis of COVID-19 [12, 13]. Numerous molecules in the immune system lead to inflammation, and the formation of inflammasomes and inflammatory cytokines are essential in these processes [14, 15].

Our previous research had indicated aberrant expression of inflammasome-related genes [16] as well as Toll-like receptors (TLRs) [17, 18] in the nasopharyngeal epithelial cells from COVID-19 subjects that might be of importance in determining the intensity of the disease’s severity. In this study, the expression of NLRP1, NLRP3, and ASC and their association with serum level of IL-1β were explored in COVID-19 patients. The results exhibited no significant differences between serum IL-1β levels in COVID-19 patients and the HS group. Additionally, the ASC and NLRP1 gene expression were increased significantly in COVID-19 patients compared to HS, whereas there was no significant increase in the expression of NLRP3 between the investigated group. The results showed a significant and negative correlation between neutrophil and lymphocyte count in patients with COVID-19. There was also a remarkable and positive correlation between the duration of hospitalization and the expression of NLRP3. Also, there was a significant and positive association between the expression of ASC and lymphocyte count, while the correlation between the expression of ASC and neutrophil count was negative. The upregulated ASC inflammasome in both nasopharyngeal epithelial cells and blood cells highlights the dysregulation of the immune response in COVID-19. This dysregulation might extend beyond the respiratory tract and contribute to systemic complications seen in severe cases, such as multi-organ dysfunction.

Numerous studies have shown that inflammation develops in patients with SARS-CoV-2 infection and leads to severe inflammation in these patients [14, 19, 20]. It has been shown that following internalization of SARS-CoV-2, translation and RNA replication of structural proteins including N, S, M, and E as well as open reading frame 3a (ORF3a), ORF8b could be initiated in the infected host cell. Among these proteins, the E protein is responsible for calcium (Ca^2+^) release from the Golgi complex, resulting in activation and formation of the inflammasome [14]. Furthermore, ORF3a interacts with TNF receptor-associated factor 3 (TRAF3), ubiquitinating ASC, and ORF8b interacts with NLRP3 to activate inflammasome and pyroptosis. Following the activation of inflammasome and pyroptosis, active form IL-1β release via gasdermin (GSDM-D-N) cell pore and a large amount of H_2_O and sodium molecules enter the cell, leading to cell swelling and pulmonary edema [21]. ORF3a can also play as a potassium (K^+^) channel in the infected cell membrane, resulting in ionic imbalance. ORF3a can also stimulate inflammasome formation and activation through reactive oxygen species (ROS) overproduction [22, 23].

It has been demonstrated that NLRP3 inflammasome can play a crucial role in viral infection pathogenesis. Stimulation of the inflammasome is probable to participate in cytokine storm formation [24]. Also, the NLRP3 inflammasome can increase SARS-CoV-2 infected elderly patients’ lethality [25]. The findings of the present study showed that there was no significant alteration in the expression of NLRP3 in the studied groups. This may be due to one of the mechanisms by which the virus SARS-CoV-2 from the host immune system can disrupt NLRP3 inflammasome activation and function by inducing a type of programmatic cell death called PANoptosis [26]. Moreover, due to the positive association between NLRP3 expression and length of hospital stay, it is probable that NLRP3 expression increases as the disease worsens [27].

A study reported that viral proteases as essential enzymes for virus replication could cleave and activate NLRP1. However, it is not clear how the inflammasome is activated following a viral infection and NLRP1 activation [28]. In this context, this study’s findings showed that in patients with SARS-CoV-2 infection, the expression of the NLRP1 and ASC genes were significantly up-regulated compared to the HS group. This study also showed that the serum level of IL-1β was not significantly increased in patients with COVID-19 than in the HS group. Consistent with these findings, a study in this field showed that only levels of IL-6, IL-8, and TNF-α showed an increase in COVID-19 patients compared to the control group [29]. These results suggest that the role of other inflammatory cytokines, such as IL-6, maybe more than that of IL-1β in causing cytokine storms [30]. Furthermore, the quantities of some inflammatory cytokines, such as IL-1β and IL-18 released during SARS-CoV-2 infection could be remarkably changed in the absence of GSDMD [26]. However, given the pleiotropic effects of cytokines and their network, this is not precisely provable [29].

The upregulation of NLRP1 and ASC in blood samples from COVID-19 cases suggests a potential role for these inflammasome components in the immune response to SARS-CoV-2 infection. NLRP1 and ASC are critical components of the inflammasome complex, which plays a central role in initiating the maturation and release of pro-inflammatory cytokines, including IL-1β. The observed upregulation could signify an attempt by the immune system to counteract viral infection through heightened inflammatory responses. However, the lack of significant change in NLRP3 expression and serum IL-1β levels between COVID-19 cases and HS is intriguing. NLRP3 is another pivotal component of the inflammasome, and its involvement in various inflammatory conditions is well-documented [31, 32]. The absence of significant changes in NLRP3 expression and IL-1β levels might reflect the complexity of the immune response in COVID-19. Other factors, such as the timing of sample collection, individual variability, or the interplay of different immune pathways, could contribute to these results. COVID-19 is known for its clinical heterogeneity, ranging from asymptomatic cases to severe respiratory distress. The varying immune responses across different patient groups could potentially explain the differential upregulation of inflammasome components. Understanding the factors that contribute to this heterogeneity is crucial for deciphering the immune dynamics during SARS-CoV-2 infection. The absence of significant differences in serum IL-1β levels challenges the notion that IL-1β is the sole driver of inflammation in COVID-19. This could imply the involvement of other cytokines or immune pathways that contribute to the observed inflammatory responses. Unraveling these alternative pathways could deepen our understanding of the disease and provide additional targets for therapeutic interventions.

The present study’s outcomes also exhibited an inverse relationship between neutrophils and lymphocyte count in patients with COVID-19. As the number of neutrophils increased, the number of lymphocytes decreased significantly, which is consistent with the results of other studies in this field [33, 34]. These findings indicated that neutrophilia and lymphopenia at hospital admission are accompanying by poor clinical outcomes in COVID-19 patients.

## Conclusion

Collectively, according to the results of this study, it might be concluded that the activation of inflammasome in the studied patients is defective for unknown reasons, and perhaps the uncontrolled inflammation in these patients is due to act of other inflammatory cytokines, which need to be investigated through assessment of the NLRP1, NLRP2, NLRP3, NLRC4, and AIM2 protein expression levels along with measurement serum levels of IL-6, IL-8, TNF-α in patients with COVID-19. The complex relationship between inflammasome components and cytokine levels in COVID-19 warrants further investigation. Longitudinal studies, more extensive patient cohorts, and mechanistic analyses could help elucidate the dynamic interactions between these factors and their contributions to disease outcomes.

## Data Availability

Data are available from the corresponding author upon reasonable request.
